# Coactivators p300 and PCAF physically and functionally interact with the foamy viral *trans*-activator

**DOI:** 10.1186/1471-2199-5-16

**Published:** 2004-09-06

**Authors:** Helmut Bannert, Walter Muranyi, Vasily V Ogryzko, Yoshihiro Nakatani, Rolf M Flügel

**Affiliations:** 1 Department of Retroviral Gene Expression, German Cancer Research Center, Applied Tumor Virology, Heidelberg, Germany; 2 Abteilung Virologie, Hygiene-Institut, Universität Heidelberg, 69120 Heidelberg, Germany; 3 André Lwoff Institut, CNRS UR079, 7 Rue Guy Moquet, Villejuif 94801, France; 4 Dana-Farber Cancer Institute, 44 Binney Street, Harvard Medical School, Boston, MA 02115, USA

## Abstract

**Background:**

Foamy virus Bel1/Tas *trans*-activators act as key regulators of gene expression and directly bind to Bel1 response elements (BRE) in both the internal and the 5'LTR promoters leading to strong transcriptional *trans*-activation. Cellular coactivators interacting with Bel1/Tas are unknown to date.

**Results:**

Transient expression assays, co-immunoprecipitation experiments, pull-down assays, and Western blot analysis were used to demonstrate that the coactivator p300 and histone acetyltransferase PCAF specifically interact with the retroviral *trans*-activator Bel1/Tas *in vivo*. Here we show that the Bel1/Tas-mediated *trans*-activation was enhanced by the coactivator p300, histone acetyltransferases PCAF and SRC-1 based on the crucial internal promoter BRE. The Bel1/Tas-interacting region was mapped to the C/H1 domain of p300 by co-immunoprecipitation and pull-down assays. In contrast, coactivator SRC-1 previously reported to bind to the C-terminal domain of p300 did not directly interact with the Bel1 protein but nevertheless enhanced Bel1/Tas-mediated *trans*-activation. Cotransfection of Bel1/Tas and p300C with an expression plasmid containing the C/H1domain partially inhibited the p300C-driven *trans*-activation.

**Conclusions:**

Our data identify p300 and PCAF as functional partner molecules that directly interact with Bel1/Tas. Since the acetylation activities of the three coactivators reside in or bind to the C-terminal regions of p300, a C/H1 expression plasmid was used as inhibitor. This is the first report of a C/H1 domain-interacting retroviral *trans*-activator capable of partially blocking the strong Bel1/Tas-mediated activation of the C-terminal region of coactivator p300. The potential mechanisms and functional roles of the three histone and factor acetyltransferases p300, PCAF, and SRC-1 in Bel1/Tas-mediated *trans*-activation are discussed.

## Background

**Figure 1:**
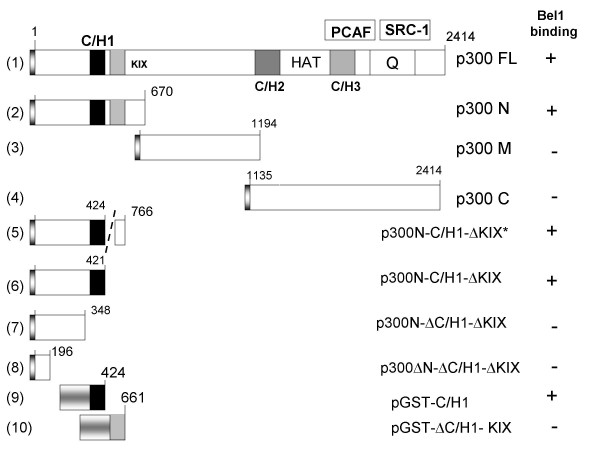
**Coactivator p300 enhances Bel1/Tas-mediated *trans*-activation. **Simplified schematic drawing of p300 domains and structure of deletion mutants (not drawn to scale). Characterized domains of p300 [2, 5] relevant for this report are highlighted by differently marked boxes: the FLAG epitope upstream of the N-terminus of p300 is marked by shaded boxes in constructs no. 1 through 8, the C/H1 and KIX domains by black and striped boxes, respectively. Numbers above the rectangles indicate the number of amino acids of p300 and its derivatives. The intrinsic HAT domain of p300 is depicted between the C/H2 and C/H3 domains; additional HATs PCAF and SRC-1 reported to associate with the distinct p300 regions [8, 24, 25] are indicated as rectangles above p300. The domain marked "Q" is the glutamine-rich domain of p300. Construct no. 5 has a deletion between the C/H1 and KIX domain (broken line). Constructs no. 9 and 10 contain the GST-C/H1 and GST-ΔC/H1-KIX fusion proteins; partial shadings mark GST regions.

In the sequential model of transcriptional regulation including viral *trans*-activation, coactivators CBP/p300 require concerted action of multiple protein factors provided the nucleosomal structures allow access to the DNA template [1-3]. The factors that interact with coactivators encompass sequence-specific DNA binding activators, non-DNA binding coactivators, and essential components of the basal transcriptional machinery. Two large, closely related human proteins, p300 and CBP, were identified and shown to function as versatile signal integrators of many transcription factors to facilitate transcriptional activation or repression, and, in addition, as connectors of multiple transduction pathways. Both proteins contain several conserved domains that include three Cys/His-rich (C/H1, C/H2, and C/H3) domains, the histone acetyltransferase (HAT), KIX, and Gln-rich (Q) domains among others (Fig. 1) [2,4,5]. It is mainly due to these domains that a plethora of transcriptional activators interact with p300/CBP. Thus, p300/CBP coactivators act as a physical and functional scaffold or bridge between various cellular or viral *trans*-activators and the basal transcriptional machinery. Both proteins function by mediating positive or negative cross talk between different signaling pathways and participate in fundamental cellular processes that include embryonic development, cell growth, differentiation, and apoptosis. In addition, they can act as tumor suppressors and, last but not least, directly interact with diverse viral *trans*-activators to facilitate virus replication or viral activator-mediated transformation [6,7].

Several models have been proposed to explain transcriptional activation. Coordinated recruitment of coactivators by diverse transcriptional activators to specific promoter target sites has been shown by a collective effort of many groups [8]. According to the models, different coactivators either modify chromatin structure by altering the nucleosomal DNA thereby affecting its accessibility to DNA-binding proteins or, alternatively, form complexes with HAT activities that by acetylation of specific Lysines in histone N-terminal tails weaken interactions between DNA and the histone octamer. Moreover, the HAT activities of some coactivators acetylate non-histone substrates such as viral and cellular *trans*-activators, for instance p53 [9].

Diverse viral *trans*-activator proteins were found to interact with distinct domains of p300/CBP and PCAF. Prominent among them are the early adenoviral E1A antigen [10,11], Epstein-Barr virus protein EBNA-2 [12], and human T-cell leukemia virus oncoprotein Tax [13,14]. Several other modifications including methylation, phosphorylation, and ubiquitination lead to either diminished or increased DNA binding of the activators that, in turn, will result in either a repression or activation of gene expression. In addition, both coactivators were reported to interact with additional HAT enzymes, namely PCAF and SRC-1 [15,16].

The apparently nonpathogenic primate foamy viruses (PFV) show a wide host range and tissue tropism and have been developed into vectors that efficiently transduce SCID-repopulating cells [17]. The PFV Bel1/Tas protein has been characterized as a transcriptional *trans*-activator of the acidic class and is known to directly interact with its responsive elements (BRE) [18,19]. Bel1/Tas is a nuclear protein and acts as the key regulator absolutely required for virus replication. The minimal Bel1-specific DNA target site is 27 base-pairs long and located within the internal promoter (IP.BRE) upstream of the second cap site that is part and parcel of the second PFV transcription unit [20]. Additional Bel1/Tas DNA target sites in the LTR region of the PFV DNA genome were not analyzed in this study. The acidic *trans*-activation domain (TAD) was mapped to the C-terminus of Bel1 with little if any protein homology to other FV Bel1/Tas proteins from different species. Previously, we identified the nuclear factor 1 (NF1) as a repressor of Bel1/Tas-mediated *trans*-activation [21]. This repression was due to the fact that the specific family members NF1-C and -X interacted with parts of the IP.BRE and its flanking sequences. Since the NF1-mediated repression of the promoter of mouse mammary tumor virus was abrogated by distinct coactivators [22], we investigated which of the known coactivators and HAT proteins were capable of interacting with the PFV Bel1/Tas activator in the context of the IP.BRE promoter that is absolutely required for virus replication [20]. Here we report that the Bel1/Tas DNA binding protein functionally interacted with p300 and with the well-known HAT factor PCAF. In addition, SRC-1 enhanced Bel1/Tas *trans*-activation. This is the first time that these cellular coactivators have been shown to interact with the Bel/Tas1 trans-activator protein. Furthermore, Bel1/Tas binding to the C/H1 domain of p300 and coactivator-driven *trans*-activation seem to follow a unique pathway.

## Results

### Coactivator p300 enhances Bel1/Tas-mediated activation

**Figure 2:**
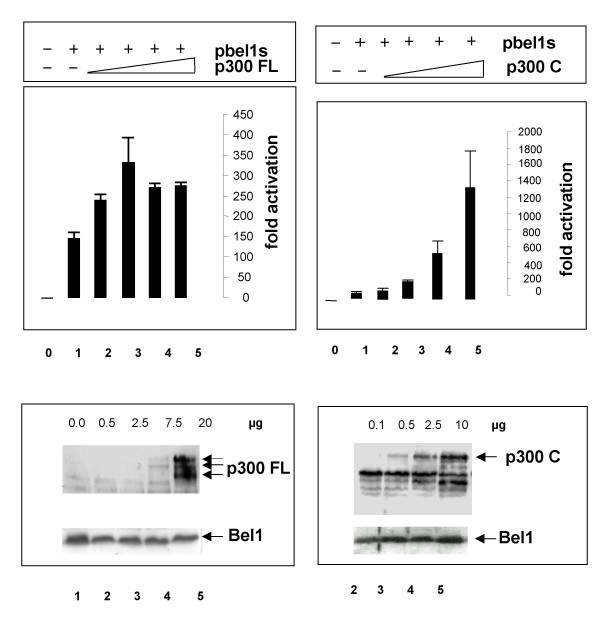
**Coactivator p300 enhances Bel1/Tas-mediated activation**. Transient expression assays were performed with pGL3-luc plasmids containing the internal PFV BRE promoter (-1 to -192) after transfection of the pCMV-bel1s expression plasmid alone or separate cotransfection with p300FL and p300C expression plasmids [4]. Normalized luc activities are shown as fold activation in upper panels (for details, see Methods). Expression levels of Bel1/Tas and p300 proteins after cotransfection of 293T cells with increasing p300FL and p300C DNA concentrations and pbel1s DNA of 1.0 μg. Aliquots of the cellular lysates used for luc assays shown in upper panel were in parallel subjected to immunoblot analysis with monoclonal antibody against the FLAG epitope of the p300FL protein (lower left panel), and against the p300C protein (lower right hand panel). Polyclonal antibody against Bel1 was used for Bel1/Tas expression (bottom lanes in lower panels).

**Figure 3:**
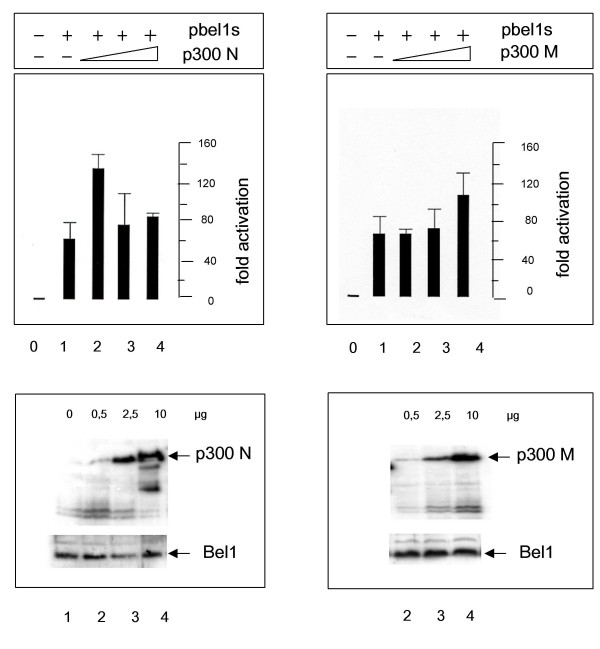
**Reporter gene assays of shortened p300 expression plasmids**. Transient expression assays were done as described under Fig. 2 except for that cotransfections were carried out with p300N and p300M plasmids; for boundaries of p300, see Fig. 1.

To examine whether p300 (Fig. 1, first line) enhanced the ability of Bel1 to *trans*-activate the Bel1 internal promoter (IP.BRE), transient reporter gene assays were performed. The IP.BRE that extends from -1 to -192 of the second cap site of the PFV genome was cloned into the pGL3-pro-luc reporter plasmid [21]. The results of the luc assays showed that full-length p300FL enhanced Bel1/Tas-mediated activation in an apparently nonlinear fashion (Fig. 2, upper left panel). To monitor the expression level of the p300 protein, Western blot analysis was carried out in parallel with increasing concentrations of the coactivator at fixed concentrations of the pbel1s expression plasmid that carries the retroviral *trans*-activator under the control of the CMV-IE promoter. The results of immunoblotting shown in the lower left panel of Figure 2 revealed that Bel1/Tas was expressed at similar levels, as expected, and p300FL expression levels proportional to the input. In parallel experiments, truncated p300 forms, p300N and p300M, were also assayed and yielded moderate levels of enhancement of Bel1/Tas-mediated activation lower than those of p300FL (Fig. 3). Unexpectedly, the most extensive level of enhancement of Bel1/Tas-induced activation was reached with the C-terminal region p300C (Fig. 2, upper right hand panel). Again, Western blot analysis of p300C showed that expression levels of p300C protein increased proportionately to the transfected plasmid DNA while Bel1/Tas expression levels were unchanged (Fig. 2, lower right panel). The precise boundaries of the three p300 versions used are shown in Figure 1. The p300 bands marked by arrows are likely due to modified p300 proteins that are known to be modified by phosphorylation, acetylation and sumoylation (2, 5, 26). We next sought to determine whether the enhancing effect of p300 on Bel1/Tas-mediated activation was due to a physical interaction with the Bel1/Tas protein.

### Bel1/Tas interacts with p300 *in vivo*

**Figure 4:**
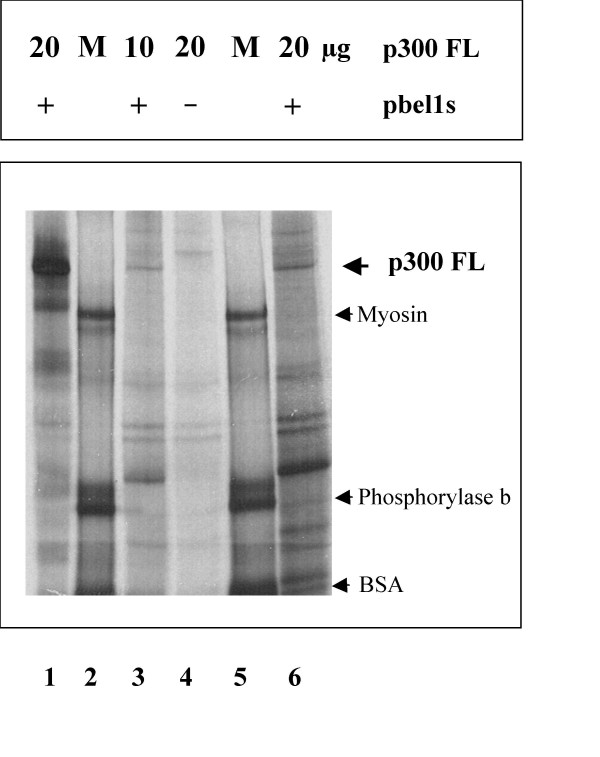
**Direct physical interaction of Bel1/Tas with p300 determined by co-immunoprecipitation**. Bel1/Tas binds to p300 *in vitro *and *in vivo*. After transfection of 293T cells with expression plasmids pbel1s and p300FL, cells were labeled with [^35^S]-Methionine plus [^35^S]-Cysteine. Cellular extracts were precipitated, separated by SDS-PAGE and exposed. Protein p300FL is marked by bold arrowhead (lanes 1, 3, and 6). In the control, pbel1s was omitted (lane 4). Arrowheads indicate the following protein size markers (M, lanes 2 and 5): myosin of apparent molecular mass of 236, phosphorylase b of 97, and BSA of 66 kDa.

To examine whether the coactivator p300 interacts with the retroviral activator Bel1/Tas, binding of p300FL to the Bel1/Tas protein was analyzed by immune precipitation. 293T cells were cotransfected with the full-length p300FL and pbel1s expression plasmids and metabolically labeled with [^35^S]-Methionine and -Cysteine. Cellular lysates were precleared and subjected to immunoprecipitation with an antibody directed against the Bel1/Tas protein except for that in lane 1 (Fig. 4). After separation by SDS-PAGE and exposure, the resulting autoradiogram showed that the Bel1-specific antibody had effectively precipitated the p300FL-Bel1/Tas protein complex at both p300FL DNA concentrations of 10 μg (Fig. 4, lane 3) and 20 μg (lane 6). In the control where pbel1s was omitted the immune precipitation did not reveal any band comparable in size to p300 (lane 4). In the immunoprecipitation shown in lane 1 instead of an antibody against Bel1/Tas, an antibody against p300 was used and showed that the bands marked in Fig. 4 was p300 (lane 1).

**Figure 5:**
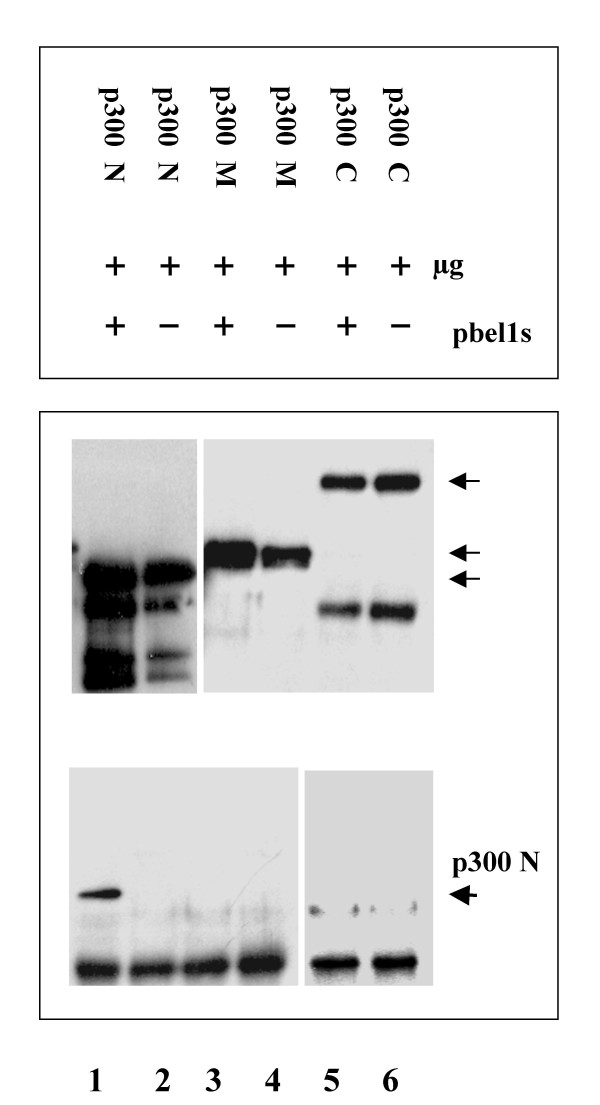
**Analysis of physical interaction of Bel1/Tas with truncated forms of p300. **After cotransfection of 293T cells with 2 μg pbel1s, and, separately with 2 μg each of p300N, p300M, and p300C expression plasmids, cellular extracts were prepared in parallel, divided into equal parts, and analyzed by co-immunoprecipitation and Western blotting. Lysates were precipitated with anti Bel1/Tas antibody and the Western blot was developed with a monoclonal anti-FLAG antiserum. The three arrows in the middle panel mark the correct sizes of the p300N, p300M, and p300C proteins (two blots pasted together). Controls for each p300 plasmid without pbel1s are in lanes 2, 4, and 6. Lower part confirms that the p300N protein specifically binds the Bel1/Tas protein (lane1, marked by arrow); two blots pasted together.

To check the data obtained, co-immunoprecipitations were performed with non-labeled 293T cells followed by Western blot analysis. Cellular lysates were prepared from 293T cells separately cotransfected with pbel1s and each one of the three truncated p300 expression plasmids, p300N, p300M, or p300C and analyzed as described above. A polyclonal antibody directed against the Bel1/Tas protein was used in the co-immunoprecipitation followed by immunoblotting with a monoclonal antibody directed against the FLAG epitope fused in-frame with the N-terminus of the three truncated p300 versions. The results shown in Figure 5 indicate that p300N protein specifically interacted with Bel1/Tas (lane 1) whereas the middle and C-terminal regions, p300M and p300C, respectively, do not seem to bind to Bel1/Tas under the conditions used (Fig. 5, lanes 3 and 5). This experiment was repeated several times and yielded the same result. In the controls, pbel1s was omitted in the cotransfections (lanes 2, 4, and 6).

### Mapping of the p300-Bel1/Tas interaction domain

**Figure 6:**
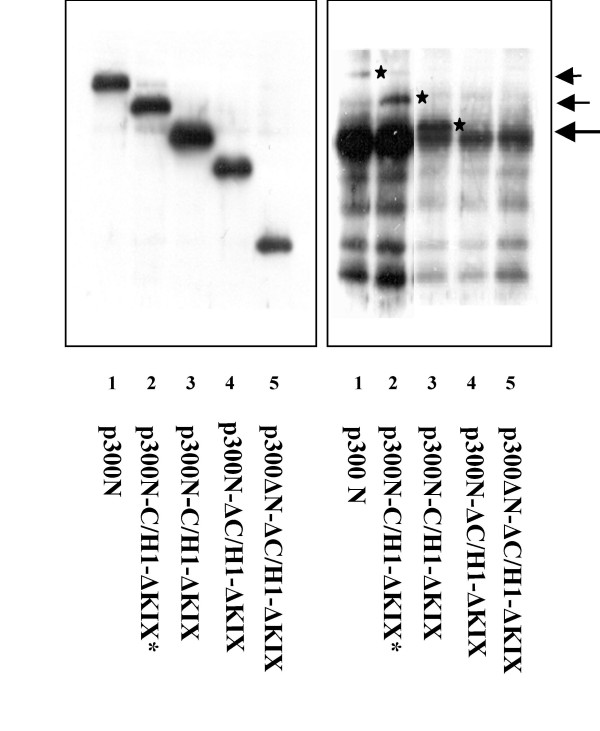
**Mapping of the p300 Bel1/Tas-interaction domain**. The p300 expression plasmids, constructs no. 2, and 5 to 8 shown in Fig. 1 were analyzed by immunoprecipitation as described above for p300N. The right hand panel shows the results of immunoblotting obtained after reaction with the monoclonal antibody against the FLAG epitope; arrows and asterisks mark the Bel1/Tas-interacting protein bands from cellular extracts of pbel1s-cotransfected cells (lanes 1–3); intentionally overexposed to visualize the marked bands in lanes 1 and 2. The left panel presents the Western blots of the five recombinant p300N plasmids used for separate co-immunoprecipitations. Numbers in brackets refer to Fig 1.

We next determined which N-terminal p300 domain was responsible for the specific interaction with the Bel1/Tas protein. Different truncated versions of p300N (Fig. 1) were prepared, cloned, and subjected to separate immunoprecipitations and Western blot analyses as described above for p300N. In addition, two different GST fusion proteins that contained either the C/H1 or KIX domain were bacterially expressed, purified, and analyzed (Fig. 1). Two different expression plasmids p300N-C/H1-ΔKIX that lacked the KIX but still expressed the C/H1 domain were capable of binding Bel1/Tas (Fig. 6, right panel, lanes 2 and 3). In contrast, the results shown in Fig. 6, lanes 4 and 5, revealed that the plasmid p300-ΔC/H1-ΔKIX that lacks both the C/H1 and KIX domains but retains the short N-terminal region of 196 amino acids did not bind Bel1/Tas. The expression levels of the five constructs were monitored by immunoblot analysis and exhibited the expected bands of p300-derived proteins (Fig. 6, left panel).

**Figure 7:**
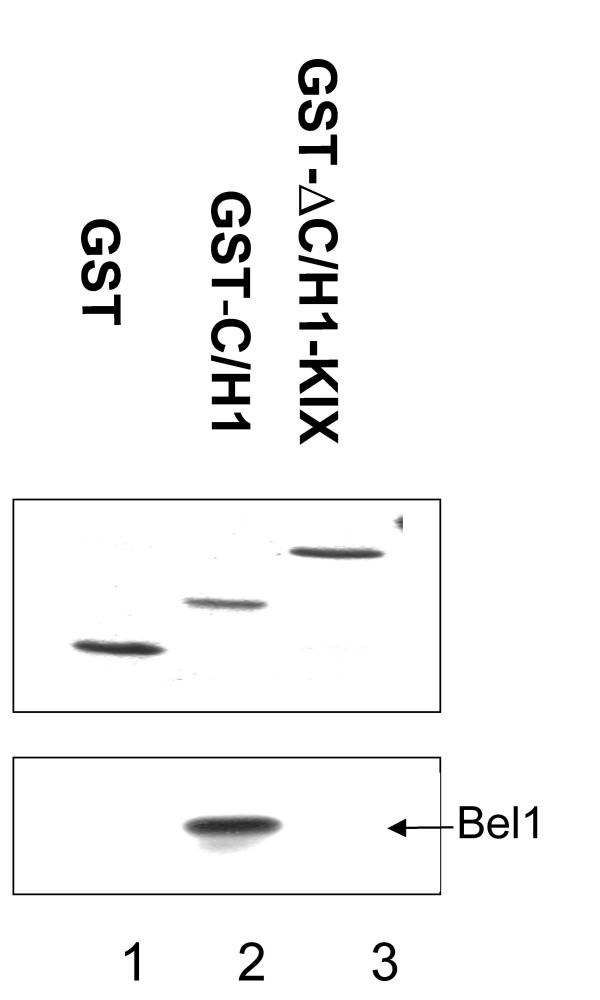
**Bel1/Tas interacts with GST-C/H1 fusion proteins detected by GST pull-down assays**. The recombinant GST-C/H1 and GST-ΔC/H1-KIX proteins were expressed in *E. coli *BL21 cells and purified by binding to glutathione Sepharose 4B. Each GST-fusion protein bound to glutathione Sepharose 4B was separately mixed with lysates obtained from pbel1s-transfected 293T cells. After incubation and extensive washing with the binding buffer, bound proteins were eluted, separated by SDS-PAGE, and visualized by staining (upper panel); immunoblotting was carried out with an antibody against Bel1/Tas protein (lower panel).

To unambiguously demonstrate that the C/H1 domain of p300 is the Bel1/Tas-interacting region, we performed pull-down assays with two pGST-C/H1 and pGST-ΔC/H1-KIX fusion proteins (Fig. 1, constructs no. 9 and 10). The results revealed that purified pGST-C/H1 clearly interacted with Bel1/Tas as shown in Fig. 7, lane 2 whereas the pGST-ΔC/H1-KIX and a control GST plasmid did not (lanes 1 and 3).

To summarize this part, our data show that p300 physically interacted with Bel1/Tas *in vivo*, and that the C/H1 domain of p300 was responsible for this interaction at least *in vitro*.

### Effect of the Bel1-C/H1 domain on Bel1/Tas-mediated activation by p300C

**Figure 8:**
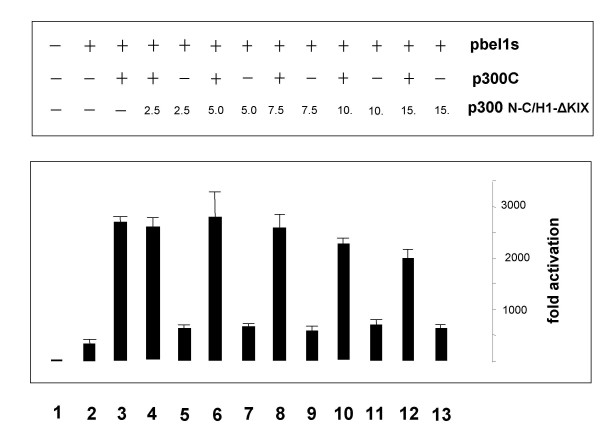
**Effect of p300-C/H1 domain expression on enhancement of p300C-mediated *trans*-activation. **Transient expression assays with pGL3-luc containing the internal BRE promoter after cotransfection of the p300C and pC/H1 expression plasmids with pbel1s.

To gain more insight into the mechanism of the C/H1-Bel1 complex that affects3 p300C-mediated activation, cotransfections of pbel1s and p300C with expression plasmid p300N-C/H1-ΔKIX were carried out (Fig. 1, construct 6). Cellular lysates of 293T cells were prepared and luciferase assays performed. Cotransfections of fixed concentrations of the pbel1s, 0.5 μg, with the pC/H1-ΔKIX expression plasmid did not enhance p300C-mediated *trans*-activation (Fig. 8, lanes 5, 7, 9, 11, and 13). In contrast, the expression of the C/H1 domain resulted in a partial suppression of the p300C-driven activation at higher C/H1 domain concentrations (Fig. 8). Similar degrees of inhibition were obtained when lower Bel1/Tas concentrations were used. Western blot analysis was carried out in parallel with increasing concentrations of the coactivator to ascertain Bel1/Tas expression (data not shown). The inhibition by the C/H1 domain explains why the level of *trans*-activation of p300N and p300FL did not reach the full extent of p300C-driven activation.

### PCAF interacts with Bel1/Tas

**Figure 9:**
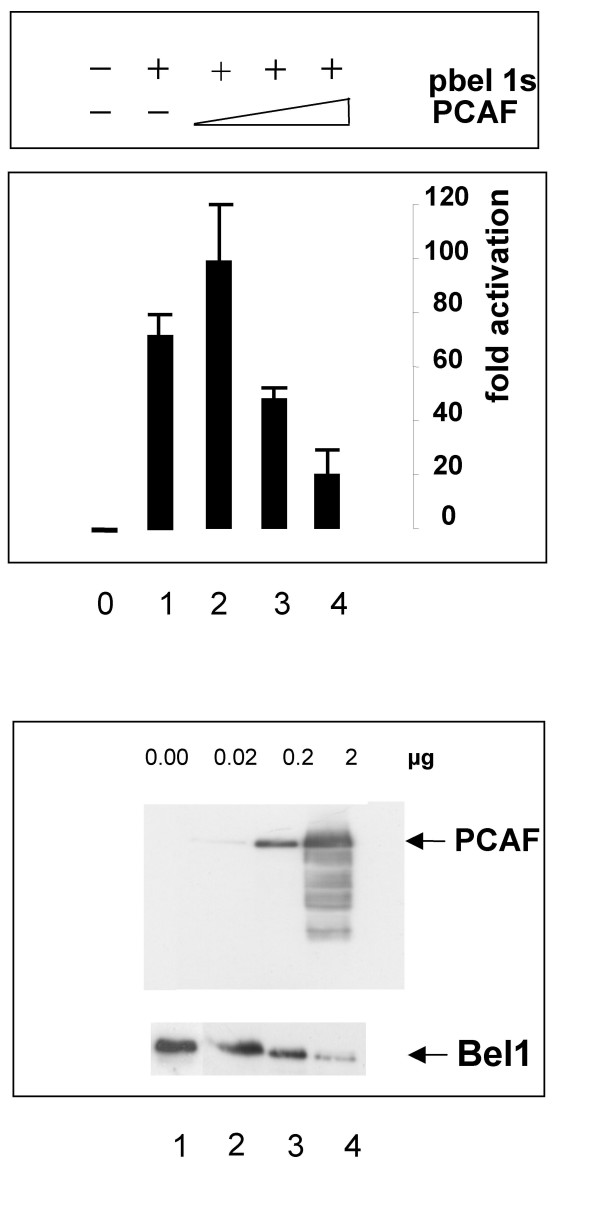
**Functional and physical interaction of Bel1/Tas with PCAF**. *A*, Transient expression assays with pGL3-luc containing the internal BRE promoter after cotransfection of the pCI-FLAG-PCAF with 0.5 μg pbel1s expression plasmid (upper panel). Aliquots were subjected to Western blot analysis (lower panel)

We next analyzed whether different HAT-expressing genes such as GCN5, PCAF, and SRC-1 were able to enhance and interact with Bel1/Tas. Transient luciferase gene assays with pGCN5 expression plasmids did not affect Bel1/Tas-mediated activation (data not shown). In contrast, when the HAT PCAF expression plasmid was used for co-expression, an enhancement of Bel1/Tas-induced activation was detectable at a concentration of 0.02 μg PCAF DNA and 0.5 μg pbel1s (Fig. 9, upper panel). At higher PCAF DNA concentrations, repression of Bel1/Tas-mediated activation was observed. When the levels of PCAF and Bel1/Tas protein expression was determined by Western blot analysis, a decreased level of the Bel1/Tas expression was detected that was likely due to degradation (Fig. 9, lower panel). The band of the PCAF protein corresponded to the correct size of about 95 kDa (marked by arrow).

**Figure 10:**
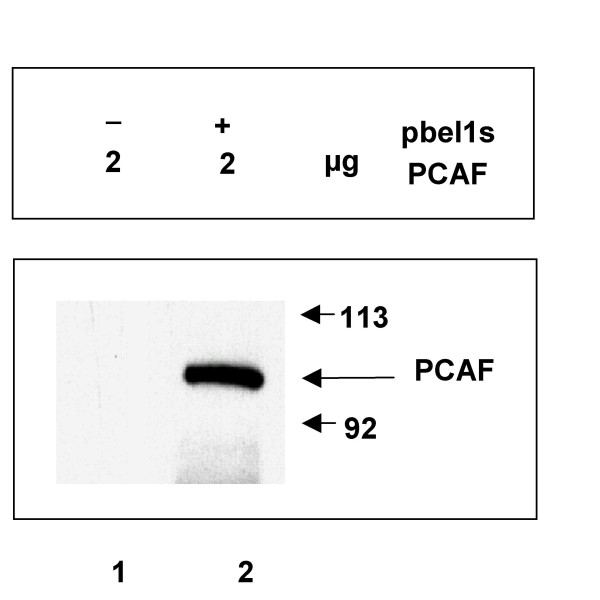
**Physical interaction of PCAF with Bel1/Tas. **Co-immunoprecipitation and immunoblot analysis of pCI-FLAG-PCAF-cotransfected 293T cells with pbel1s DNA (lane 2). The cellular lysates were incubated with an anti Bel1/Tas antibody and treated as described in the legend to Fig. 5 except for that an antibody directed against the FLAG epitope of PCAF was used for immunoblotting. In the control, cotransfection was done without pbel1s (lane 1).

We next analyzed the potential interaction between the Bel1/Tas and PCAF proteins by carrying out co-immunoprecipitation with 293T cellular lysates that had been cotransfected with 2.0 μg pbel1s and pCI-PCAF expression plasmids, followed by immunoreaction with an antibody against Bel1 and subsequent immunoblotting with an anti-FLAG antibody to detect PCAF. The results showed that PCAF did indeed interact with the PFV Bel1/Tas activator (Fig. 10, lane 2). As control, an immunoprecipitation and Western blot analysis was performed with the PCAF plasmid in the absence of pbel1s (Fig. 10, lane 1). The result showed that a PCAF band was not detectable under the conditions used.

**Figure 11:**
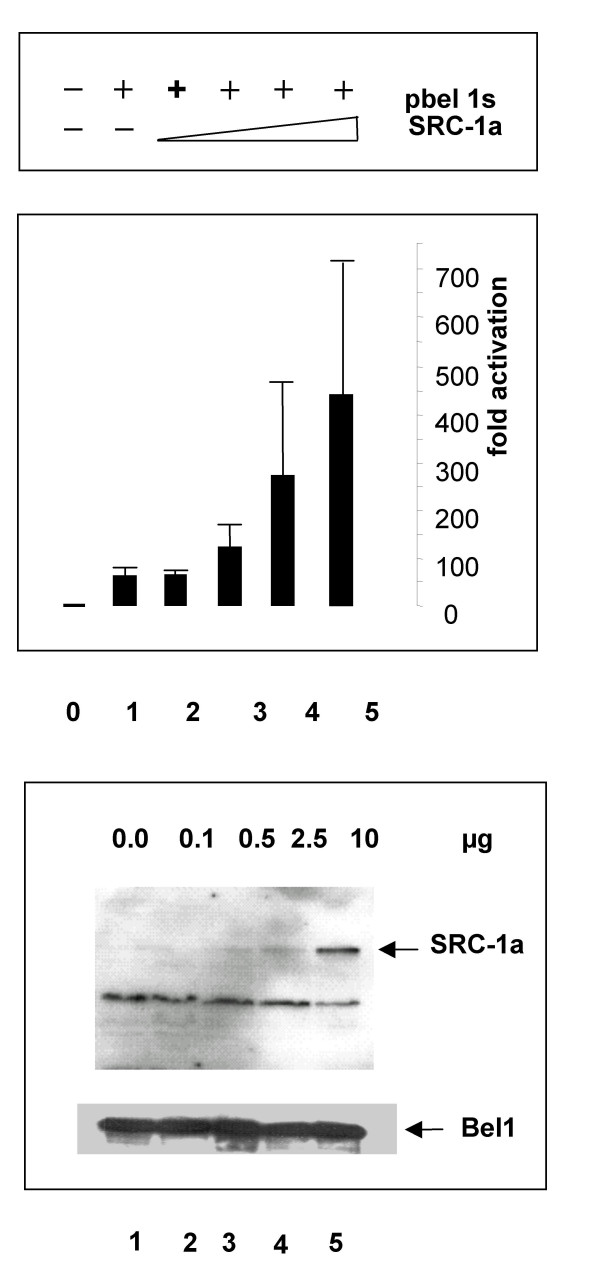
**Coactivator SRC-1 enhances Bel1/Tas-mediated *trans*-activation**. Reporter luc gene expression assays with pGL3-luc plasmids containing the internal PFV BRE promoter after cotransfection of pCR3.1-FLAG-SRC-1a with the pbel1s expression plasmid (upper panel). Immunoblotting of 293T cellular extracts cotransfected with the SRC-1a and pbel1s expression plasmids (lower panel).

To determine if an additional HAT protein, SRC-1, affected Bel1/Tas-mediated activation, transient reporter gene expression assays were carried out after cotransfection of 293T cells with the coactivator SRC-1a and 0.5 μg pbel1s. Surprisingly, a relatively strong enhancement of Bel1/Tas-mediated activation was detected (Fig. 11, upper panel). The level of expression of both SRC-1a and Bel1/Tas proteins was determined and found to be approximately proportional to the input (Fig. 11, lower panel).

To assess whether SRC-1a was able to physically interact with the Bel1/Tas protein, immunoprecipitations and Western blot analysis were carried out under different conditions. The specificity of SRC-1a was ascertained by using monoclonal antibody directed against SRC-1a in control reaction. However, evidence for an interaction between the SRC-1a protein and Bel1/Tas was not obtained.

## Discussion

It was previously reported that Bel1/Tas is capable of inducing the expression of many cellular genes [23]. While it is known that Bel1/Tas binds directly and to a large number of DNA target sites [18-21], the mechanism of activation and the identity of the cellular partner molecules of Bel1/Tas remained unknown. As a first step, we have sought to identify the cellular proteins that interact with the PFV retroviral *trans*-activator and mediate its activating potential. The data presented here show that the coactivators p300 and HAT PCAF physically bound Bel1/Tas *in vitro *and both enhanced Bel1/Tas-mediated activation whereas SRC-1 enhanced with Bel1/Tas activation without direct binding. According to our data, Bel1/Tas specifically interacted with the C/H1 domain of p300, although we cannot rule out binding to other p300 domains with much lower affinity not detectable under the rather harsh conditions of co-immunoprecipitation used here.

When the levels of the relative luciferase activity of p300FL and its three shortened versions are compared, it is noteworthy that p300C reached the highest level of enhancement of Bel1/Tas-mediated activation (Fig. 2). Besides its intrinsic HAT activity, p300C contains both the intact C/H3 and Q domains that interact with the HAT enzymes PCAF and SRC-1, respectively [10,24,25]. These three HAT enzymes are likely responsible for the large enhancement of the observed *trans*-activation either directly by acetylation of Bel1/Tas or indirectly by histone acetylation, or both. In contrast to p300C, p300N and p300M do not possess any HAT activities nor do they bind to HAT-containing interaction partners. On the other hand, it is well known that p300 and its three subregions bind a plethora of various partner molecules leading to either activation or repression of transcription. Since the high level of p300C-mediated activation was partially inhibited in cotransfections with C/H1 and pbel1s, competition for Bel1/Tas between the C/H1 and the p300C-terminal interaction partners PCAF and SRC-1 cannot be ruled out so that both direct and indirect mechanisms might be responsible for the relative increase in Bel1/Tas-mediated activation by p300C. When Bel1/Tas binds to the C/H1 domain, the degree of Bel1/Tas acetylation may be much lower, since the protein surface of Bel1/Tas may be occluded and, hence less accessible. Some residual Bel1 acetylation might still occur by endogenous p300 and PCAF. Other factors might play additional roles. The ability of full-length p300 to *trans*-activate Bel1/Tas was relatively low for two reasons. First, the transfection efficiency of the full-length p300 is very low because of its large plasmid size, and the concentration of endogenous p300 is limiting. Secondly, the activation loop of p300 HAT is not fully activated by auto-acetylation as required for full *trans*-activation [26].

Since the relative activation by SRC-1 was not as high as that of p300C, we consider the HAT activity of PCAF as one of the major players of Bel1/Tas-mediated *trans*-activation. This result is supported by the observed enhancement of Bel1/Tas activation after cotransfection with PCAF that resulted in higher levels of Bel1 acetylation thereby leading to increased binding to the IP.BRE [J. Bodem, personal communication]. Thus, the observed high level of enhancement of p300C might be due to the synergistic effects brought about by formation of ternary p300C-PCAF-Bel1 and binary p300C-SRC complexes, respectively. In these multimeric protein complexes, Bel1/Tas binds p300C indirectly through PCAF. Consistent with the HAT activities of PCAF and p300, we detected acetylated Bel1/Tas in pbel1s-transfected 293T cells using monoclonal antibody against acetyl-Lysine after immunoblotting (our unpublished data). It is intriguing that the distribution of the closely spaced Lysines of Bel1/Tas apparently mimics the correspondingly spaced Lysines in histones. This observation is further complicated by our observation that cotransfection with higher levels of PCAF led to a reduced stability of Bel1/Tas (Fig. 9). We assume that PCAF acetylates or even hyper-acetylates the Bel1/Tas protein at closely spaced Lysines in analogy to other activators reported previously [9]. The decreased stability of acetylated Bel1/Tas might indicate that modified Bel1/Tas is less stable than the unmodified form. This observation adds an additional layer of combinatorial regulation to Bel1/Tas-mediated *trans*-activation.

It is intriguing that some viral *trans*-activators interact with more than a single p300 domain [10,14]. However, Bel1/Tas might recruit a second interacting region of p300 through binding PCAF (Fig. 10) that is known to interact with a p300 domain different from the C/H1 domain (Fig. 1) [10]. The complex nature of p300-Bel1/Tas interactions reported here might serve to strengthen the overall binding affinity between Bel1/Tas and the PCAF interaction domain of p300 within a larger transcriptional complex [27]. PCAF is known to specifically acetylate distinct Lysine residues of a subset of core histones and thereby regulate the transcriptional activity of many genes depending on the genetic context. It is well documented that acetylation, methylation and other covalent histone modifications are essential signals for the regulation of transcription [28].

It remains to be seen whether the stronger level of enhancement of p300C is a special if not unique feature of Bel1/Tas activation and due to over-expression or to repressive effects of other p300-interacting protein factors that cannot bind to the truncated p300 protein. Alternatively, many other factors were reported to bind to the C-terminal domains of p300 that also encompass general transcription factors TBP and TFIIB proteins that might also be responsible for the enhancement observed here [1-3].

In search of viral and cellular activators that are comparable with the ability of Bel1/Tas to interact with the C/H1 domain of p300, we found one case. A report indicates that EBNA-2 protein shares many features with Bel1/Tas that include the C-terminal acidic activation domain as well as the abilities to bind both the C/H1 domain and PCAF [12]. There remain two differences, however. First, EBNA-2 binds to both the C/H1 and the C/H3 domain, and, secondly, PCAF does not coactivate EBNA-2 in strong contrast to Bel1/Tas [12].

Of note, an additional HAT enzyme, GCN5, did not interact with Bel1/Tas when tested in reporter genes assays indicating that only distinct HAT sets such as those identified in this report specifically interact with Bel1/Tas in *trans*-activation. The precise roles of the HAT activities of PCAF, p300, and SRC-1 during Bel1/Tas-mediated *trans*-activation remain to be addressed in future studies.

## Conclusions

Coactivators PCAF and p300 were identified to physically and functionally interact with the spumaviral Bel1/Tas *trans*-activator. Coactivator SRC-1 was found to strongly enhance Bel1/Tas-mediated *trans*-activation. The C/H1 domain of p300 was responsible for binding the retroviral activator and found to partially inhibit the p300-driven *trans*-activation.

## Methods

### Antibodies

Mouse monoclonal antibodies directed against the FLAG epitopes of p300FL, p300N, p300M, p300C, and PCAF were purchased from Sigma, rabbit polyclonal antibodies against the N-terminal p300FL, the C-terminal p300C, and SRC-1a from Santa Cruz Biotechnology. The polyclonal serum direct against Bel1/Tas was used as described previously [23]. Typically, 5 μl of each antiserum was used for each immunoprecipitation. Monoclonal antibody against acetyl-Lysine was purchased from Sigma.

### Plasmids, cells, transfections, and metabolic labeling

Plasmids pUC18, pCMVβ-gal, pbel1s [29], pGL3-pro-IP. BRE (-1 to -192), pCI-FLAG-p300FL, pCI-FLAG-p300N, pCI-FLAG-p300M, pCI-FLAG-p300C [4], and pCI-FLAG-PCAF [29] were separately, or in the combinations indicated, transfected into 293T cells using Lipofectamine 2000 (Invitrogen). In general, unless otherwise indicated, 0.1 – 10 μg plasmid DNA were transfected into 293T cells grown in Petri dishes with a diameter of six cm. Full-length pCI-FLAG-300FL plasmids and three different shortened versions (Fig. 1, constructs no. 1 to 4) were grown in *E. coli*, DH5α cells [4]. The PFV internal promoter was constructed by PCR-mediated amplification of the defined promoter fragments as reported previously [19,21]. Recombinant clone p300N-C/H1-ΔKIX* (construct no. 5, Fig. 1), was constructed by first generating two PCR products with pCI-p300FL as template using the sense (s) and antisense (as) primers s1: 5'-CTTATGGTTCACCATATACTCAGAATCC-3', as1: AAACTGGAACCATGCCTGCATTTCTCTTATCACC-3', s2: 5'-GAAATGCAGGCATGGTTCCAGTTTCCAT-3', and as2: 5'-GGAAGGAACTGGCCCTGGTTGGAAGGCTGTTG-3' to amplify the sequence from nucleotide 755 to 1275 and nucleotides 1984 to 2297 fused in-frame. The resulting DNA product of 833 nucleotides was cloned into the pCR2.1 Topo vector and designated as pCR2.1-ΔKIX. An *SphI-NotI *DNA fragment obtained from pCR2.1-ΔKIX and was inserted into pCI-p300N that had been predigested with *Sph*I and *NotI*. The borders of construct no. 5 are shown in Fig. 1 and the expressed recombinant protein had the expected size. p300-C/H1-ΔKIX (no. 6) was constructed by inserting the *SphI*/*NotI *DNA fragment from pGEX-C/H1 (35) into pCI-p300N digested with *SphI*/*NotI *for expression of residues 1 to 424 of p300. Construct no. 7 (Fig. 1) was constructed by digesting pCI-p300N with *SphI *and re-ligating the larger fragment for the expression of residues 1 through 347 of p300. Finally, p300ΔN-ΔC/H1-ΔKIX was constructed from pCI-p300N by digesting with *MunI *and re-ligated to express residues 1 to 196 of p300. Bacterial plasmids coding for glutathion-S-transferase (GST) fusion proteins pGST-C/H1 (328–424) and pGST-KIX (436–661) (Fig. 1) were constructed from pGEX-6p-2GST-p300 [30] and grown in *E. coli*, BL21 cells. pCR3.1-FLAG-SRC-1a was grown in *E. coli*, JM109 cells [24]. Human 293T or HeLa cells were cultivated in DMEM medium supplemented with 1% penicillin and streptomycin, 1% Glutamine and 10 % fetal calf serum.

Plasmid p300FL was transfected into 293T cells and metabolically labeled with L-[^35^S]-Methionine plus L-[^35^S]-Cysteine (spec. act. of 37 TBq/mM, PRO-MIX, Amersham) for 6 hr. Cells were harvested and used for immunoprecipitation as described above. The precipitates were analyzed by SDS-PAGE on 12% gels, dried, and exposed on KODAK Biomax MR1 films.

### Luc reporter gene expression assays

Plasmid pCMV-βgal directing β-galactosidase expression from the CMV-IE promoter was used for normalization of transfection efficiency. Luc reporter gene assays were performed and quantified as described [31] using a Luminoskan TL Plus luminometer (Labsystems, Frankfurt, FRG). pUC18 vector plasmid DNA was used as carrier DNA to equalize the DNA concentration of each transfection. Cells were harvested 18 h after transfection. The results of luc assays were based upon at least triplicate experiments on multiple independent occasions. Expression levels were monitored by Western blot analysis.

### Co-immunoprecipitation

Immunoprecipitation was performed as described previously with minor modifications [32]. Lysates of subconfluent cotransfected layer of 293T cells were prepared by first washing the cells with PBS, and subsequently lysed in lysis buffer (150 mM NaCl, 20 mM Tris-HCl [pH 7.5], 1 mM phenylmethylsulfonyl-fluoride containing 1% (v/v) Triton X-100. To inhibit unspecific protease activity, protease inhibitors (Biomol) were added to the lysis buffer. Lysates were precleared with protein A-SepharoseCL-4B (Amersham Bioscience AB, Uppsala). Co-immunoprecipitation of p300FL, p300N, p300M, p300C, SRC-1, and PCAF were performed with rabbit anti Bel1 antiserum [23]. The immune precipitates were retrieved with protein A-SepharoseCL-4B (Pharmacia) and eluted by boiling. To detect the specific precipitate, immunoblotting was performed with specific antibodies against expressed p300, shortened p300 versions, and SRC-1. For the detection of expressed PCAF, Western blot analysis with monoclonal anti-FLAG antibody was carried out. The immunoprecipitates were washed three times with the lysis buffer and analyzed by immunoblotting on 12% gels for expressed p300FL and on 14% gels for shortened p300 forms, PCAF, and SRC-1a. The experiments were repeated at least three times, specially the immunoprecipitations of lysates of p300C-transfected cells.

### GST pull-down assays

The recombinant GST-C/H1 and GST-KIX proteins were expressed in *E. coli *BL21/DE3 cells after transformation with the corresponding plasmids. Expressed proteins were purified by binding to glutathione Sepharose 4B resin. Each of the GST-fusion proteins bound to glutathione Sepharose 4B were mixed with lysates obtained from pbel1s-transfected 293T cells. After incubation at 4°C overnight in binding buffer [30] and extensive washing with the binding buffer, bound proteins were eluted, separated by SDS-PAGE, and visualized by staining with Coomassie Brilliant Blue according to Ariumi [30] or detected by Western blot analysis.

### Immunoblotting

Cells were harvested two days after transfection by lysis in 1% SDS and the protein concentration was determined using the DC protein assay (BioRad). Identical amounts of proteins were separated by SDS-PAGE on 12% gels, blotted, reacted with monoclonal serum directed against the FLAG epitope of the four different FLAG-tagged p300 (Sigma), or polyclonal serum direct against Bel1 [23], and detected by enhanced chemoluminescence.

## List of abbreviations used

CBP, CREB-binding protein; C/H1, 2, and 3, Cys/His-rich domains of p300; FL, full-length; HAT, histone acetyltransferase; LTR, long terminal repeat; luc, luciferase; PCAF, p300/CBP-associated factor; Q, Glutamine-rich domain of p300; SRC, steroid receptor coactivator; p300N, p300M, and p300C, amino-, middle and C-terminal regions of p300; PFV, primate foamy virus; IP.BRE, internal promoter Bel1 response element; TAD, *trans*-activation domain; NF1, nuclear factor 1; CMV, cytomegalovirus; GST, glutathione-S-transferase.

## Authors' contributions

HB and WM contributed equally to the manuscript. VO carried out the molecular cloning of p300 derivatives. YN participated in the design of the study. All authors read and approved the final manuscript.
